# Reputation and Reward: Two Sides of the Same Bitcoin

**DOI:** 10.3390/s16060776

**Published:** 2016-05-27

**Authors:** Sergi Delgado-Segura, Cristian Tanas, Jordi Herrera-Joancomartí

**Affiliations:** Departament d’Enginyeria de la Informació i les Comunicacions, Universitat Autònoma de Barcelona, Bellaterra, Barcelona 08193, Spain; ctanas@deic.uab.cat (C.T.); jordi.herrera@uab.cat (J.H.J.)

**Keywords:** mobile crowd sensing, cryptocurrencies, reputation systems, privacy, bitcoin

## Abstract

In Mobile Crowd Sensing (MCS), the power of the crowd, jointly with the sensing capabilities of the smartphones they wear, provides a new paradigm for data sensing. Scenarios involving user behavior or those that rely on user mobility are examples where standard sensor networks may not be suitable, and MCS provides an interesting solution. However, including human participation in sensing tasks presents numerous and unique research challenges. In this paper, we analyze three of the most important: user participation, data sensing quality and user anonymity. We tackle the three as a whole, since all of them are strongly correlated. As a result, we present PaySense, a general framework that incentivizes user participation and provides a mechanism to validate the quality of collected data based on the users’ reputation. All such features are performed in a privacy-preserving way by using the Bitcoin cryptocurrency. Rather than a theoretical one, our framework has been implemented, and it is ready to be deployed and complement any existing MCS system.

## 1. Introduction

Mobile Crowd Sensing (MCS) arises as a new sensing paradigm based on the power of the crowd jointly with the sensing capabilities of smartphones. The increasing popularity of such devices paired with the inherent mobility of their owners enables the ability to acquire local knowledge from the individual’s surrounding environment. This local knowledge ranges from location information to more specialized data, such as pollution levels, going through a longer list of personal and surrounding context, noise levels or traffic awareness, among others.

A large number of crowd sensing applications have already been developed, although typically for experimental purposes and to show the usefulness of such a sensing paradigm. For instance, Eisenman *et al.* [1] harnesses the sensing capabilities of smartphones paired with the individual’s *smartness* to determine the most “bikeable” routes in a city. Similarly, the Common Sense project [2] allows individuals to measure their personal exposure to air pollution and share it with their social sphere. On the other hand, applications such as Nericell [3] measure the interaction between individuals to infer the context within which they carry out their activities. Furthermore, using environmental sound and mobile devices’ microphones, Xu *et al.* [4] developed a system capable of counting the number of people in a place. The system could distinguish different people with no previous knowledge of them. Other systems, like the one proposed by Rosen *et al.* [5], have shown how MCS could be also used for improving WLAN performance, by periodically sampling the WiFi performance using mobile devices with a negligible battery impact.

However, MCS presents numerous and unique research challenges, most of them based on the fact that human participation is in the loop, and range from participatory and opportunistic data collection, proper incentive mechanisms, transient network communication and big data processing. Nonetheless, human participation raises singular issues regarding the privacy and security of data, as sensitive information, such as human voice or location, may be revealed. Furthermore, the quality and trustworthiness of the contributed data (e.g., counterfeit data contributed by malicious users) should also be addressed.

In this paper, the PaySense system is described, and an exhaustive security analysis is performed. The main contributions of our proposal are the following. We provide a secure general framework that can be used in any MCS application to economically reward users by paying them bitcoins. The provided system also allows one to keep track of every user’s reputation, a value that can be used for the MCS application in the data validation process. Furthermore, the system provides a high degree of user privacy since Bitcoin addresses are used as user pseudonyms, and the system takes special care in avoiding linkability threads, a well-known problem of Bitcoin transactions. Finally, the system has been implemented and is publicly available to be included in any MCS application [6].

The rest of the paper is organized as follows. In Section 2, we provide a review of the state of the art focused on three important challenges in MCS: user participation, data sensing quality and user anonymity. Section 3 introduces the Bitcoin cryptocurrency and how it can be applied for user rewarding and reputation accountability. In Section 4, we describe the PaySense system, providing detailed information on the system entities and their interactions, taking special care about users privacy. In Section 5, we perform an exhaustive security analysis of PaySense, providing a clear adversary model and detailing all of the security measures that have been developed to mitigate possible attacks. Finally, Section 6 concludes the paper and gives some guidelines for further research.

## 2. State of the Art

The structure of this section is based on the three main challenges of MCS addressed in PaySense: user participation, data sensing quality and user anonymity. The main proposals appearing in the literature are summarized, and the interrelation between those three challenges is also outlined in this section.

### 2.1. User Participation

MCS systems typically involve a very large number of users or *crowd sensors* in the sensing task by collecting and sending local data obtained through their sensor-enabled mobile devices to a data collection center. The performance and usefulness of such sensor networks heavily depends on the crowd sensor’s willingness to participate in the data collection process. Therefore, incentive mechanisms are of utmost importance in MCS scenarios to engage as many crowd sensors and to provide the data collection center with a considerable wealth of data.

Based on the nature of user participation, we can discern between two MCS paradigms, as introduced by Lane *et al.* [7]: *participatory* sensing and *opportunistic* sensing. The first sensing paradigm requires the user to have complete consciousness of what, where and when it is being sensed. For instance, it may require the users to observe and describe their surrounding environment, typically assuming a higher degree of involvement for the crowd sensors. On the other hand, in opportunistic sensing scenarios, the data is acquired in the background, namely the data are being sensed opportunistically and automatically sent (*i.e.*, without the user’s active participation) using the device’s network connection to the data collection center. Even though it may seem that users would be more willing to participate in this kind of scenario, battery wastage or large amounts of data being sent may cause the user to refuse to participate in sensing tasks.

The design of incentive mechanisms to stimulate participation has been addressed in crowd sensing scenarios [8], although similar needs were previously identified in the field of *ad hoc* or P2P networks [9], which also relay on the participant’s willingness, in that case to forward packets. The nature of the incentives provided to crowd sensors allows its classification on the following categories: *economic*, *service-based* and *social* [10,11].

The incentives mechanisms in the first category model the problem through a financial approach where crowd sensors get paid or receive some kind of credit based on the provided service [12]. However, the lack, until now, of an easy to use, inexpensive and secure micro-payment system pushes towards dealing with external entities, such as banks or financial institutions that impose too high transaction fees for a practical pay-per-sense solution.

On the other hand, service-based incentives try to foster participation by taking a user-centric approach in which feedback or individual benefits are perceived by the crowd sensors in a way that is relevant to them [13]. However, not all sensing scenarios may fall into such categories, since the sensing objective may not be of interest or provides no benefits to the crowd sensors.

Finally, gamification techniques have also been used to incentivize participation in mobile crowd sensing applications [14] in what we consider a “social” reward. However, apparently, the effectiveness of such incentive schemes depends considerably on a large set of interrelated factors from community-related (topic, number of participants, *etc.*) to cultural or motivational factors [15]. This multitude of determinants makes it difficult to assess if the gamification scheme would work in all given MCS scenarios.

### 2.2. Data Sensing Quality

In MCS systems, there is no control over the crowd sensors, and we cannot assume that all individuals will behave in the exact same manner or will be equally honest. Therefore, the overall quality of the sensor readings can see itself deteriorated if counterfeit data are received from malicious users. Hence, the obvious question is how to validate the sensing data that crowd sensors provide to the system. A commonly-used approach is to validate the data depending on the trust level of the crowd sensor that reports it [16].

Trust and reputation systems have long been studied to establish trust relations among the members of an on-line community where prior knowledge of the participants may not even be available or where the community is formed by a crowd of anonymous volunteers. In such systems, each user is provided with a reputation score that indicates his or her trustworthiness when acting as the information provider. For instance, Jøsang *et al.*, in [17], introduce several reputation quantification models, while [18] presents a reputation framework based on fuzzy logic in the context of social participatory sensing. Such reputation scores can be increased each time a crowd sensor provides valid data.

Nevertheless, a validation scheme based only on the user’s reputation as a sensing data reliability measure does not provide a useful method since, in an initial state, the system could not identify any crowd sensor as trustworthy, leading it towards a deadlock state. In order to avoid such situations, a combination of reputation systems and collective knowledge can be applied [19].

#### Data Quality *vs.* Incentive Mechanisms

Combining both reputation systems and incentive mechanisms in the context of an MCS application is especially sensitive. Given the self-interested and possibly selfish nature of individuals, there exists the possibility of crowd sensors acting in a way to maximize their own gains, regardless of the impact that this may have on the overall sensor network. With higher incentives provided for participation, the more motivated the crowd sensors will be to increase their personal benefits possibly by providing misleading information. Therefore, improving the design of an incentive mechanism should imply an improvement of the corresponding quality control process and, hence, of the reputation system.

### 2.3. User Anonymity

An important aspect of MCS scenarios is the collection of potentially sensitive information pertaining to individuals [20]. For instance, Global Positioning System (GPS) sensor readings can be used to track users’ movements and profile them for other purposes besides their crowd sensing tasks. Furthermore, if the MCS application collects “delicate” information, such as criminal acts, crowd sensors may be reluctant to provide data without proper anonymity measures for the fear of being collaterally involved in such acts. Hence, it is necessary to preserve the privacy of crowd sensors, but at the same time to ensure the usefulness of the MCS application.

A popular approach for preserving users’ privacy is to use pseudonyms when sending sensed data to the data collection center (see [21], for example). These pseudonyms are typically randomly generated and bare no relation to the individual’s real identity. In these cases, we say that individuals benefit from pseudo-anonymity, since we cannot infer their real identity from the pseudonyms, but we can still identify subsequent sensor readings as reported by the same user with the same pseudonym. Another approach is to remove any user identifying attribute from the sensing data before sending them to the data collection center [5]. Obviously, this approach can be applied only in those MCS applications that are solely interested in the actual data, and no further interaction with the crowd sensors is necessary.

However, user identification not only can be achieved by the identification of the source of the sensed information, but also with the sensed information itself. It is well known that some spatiotemporal patterns can be used to identify users in a system [22]. To avoid these anonymity threads, a privacy-preserving mechanism, like *k*-anonymity resistance, can be deployed. Such techniques have already been successfully proposed in mobile crowd sensing scenarios to reduce the risk of identification when crowd sensors provide their location [23].

#### 2.3.1. Anonymity *vs.* Incentive Mechanisms

The crowd sensor should be able to provide sensing data in an anonymous way while still perceiving incentives for that task, and at the same time, crowd sensor network authorities should be able to ensure that dishonest users cannot earn unlimited credit.

Few works can be found in the literature dealing with this problem. In [24], the authors analyze the interaction between privacy and incentive mechanisms, when anonymization mechanisms are applied over the sensed data. The analysis is focused on how privacy mechanisms affect the price of data sensing when the incentive mechanism is based on the greedy incentive algorithm [25]. However, anonymization is focused on sensed data, and there is no information on how users are identified in the system. Li and Cao [26] propose an incentive scheme where users are rewarded for their contributions with tokens (credits) that can later be exchanged for additional services or for real-world objects. Unfortunately, the incentive scheme relies on a trusted third party to ensure that the anonymity of the users is preserved by applying blind signatures and commitment techniques.

#### 2.3.2. Anonymity *vs.* Reputation Systems

An anonymous reputation system may seem an apparent paradox considering that an anonymous system requires complete unlinkability between the user’s identity and the sensed data, while reputation systems claim this link to be existent in order to maintain an updated reputation score for each user.

In [27], the authors acknowledged the importance of a privacy-preserving reputation system. While rarely explored in the context of MCS networks, this has been addressed in peer-to-peer networks, where users are allowed to create multiple pseudonyms, each with its own reputation score, to achieve anonymity [28]. Then, different pseudonyms may be used in different interactions with other entities, forcing adversaries to trace sequences of pseudonyms used by the same individual in order to reveal their identity.

Having different pseudonyms, each with its own reputation score, however, is detrimental to the reputation system, which should be continuous and applicable transversely to all pseudonyms corresponding to the same user. Miranda and Rodrigues develop this idea in [29] and provide a mechanism that allows users to transfer the reputation information from one pseudonym to another, without disclosing this link or the user’s real identity.

However, if not properly performed, reputation transfer between pseudonyms may provide a linkage mechanism. Take, for example, an individual having the highest reputation score among all pseudonyms. Then, it would be straightforward for an adversary to establish a link between this pseudonym and the pseudonym conserving the same reputation score after the transfer process. Christin *et al.* [30] discuss this issue and provide a solution in the context of participatory sensing. They also acknowledge the need of a trusted third party to ensure that anonymity is preserved in such scenarios. However, the main drawback of their proposal is that users lose reputation in favor of pseudonym unlinkability, but the lost reputation could not be recovered.

Finally, the possibility of changing pseudonyms may allow a Sybil attack, where malicious users may replicate sensor readings under different pseudonyms to earn more reputation or credit, so new measures have to be developed to enforce that every crowd sensor only has a valid pseudonym at a given time.

## 3. Bitcoins: The Integration Tool

Bitcoin is a cryptocurrency system [31] that emerged in early 2009, and it has been rapidly adopted due to its three main properties: security, decentralization and user anonymity (see [32] for detailed information on the Bitcoin system). Bitcoin system security is based on elliptic curve public key cryptography together with the help of hash functions; its one-wayness provides a way to define an easily verifiable and fine-grained adjustable proof-of-work. Furthermore, double-spending is prevented by maintaining a public non-modifiable ledger, the *blockchain*, which includes all performed transactions. On the other hand, regarding decentralization, no central authority is supposed to control the Bitcoin payment system. Instead, a distributed approach has been adopted for data storage and data transmission using the Bitcoin peer to peer (P2P) network. Such a distributed approach has different sides: data storage, data confirmation and data transmission. The core information of the Bitcoin system is included in the so-called blockchain, which is stored in every full-client node of the Bitcoin system in order to be able to validate new transactions. On the other hand, new transactions are confirmed by adding them to the blockchain through the mining process, a process that is also distributed and that can be performed by any user of the Bitcoin network using specific-purpose software (and hardware). Mining bitcoins helps to confirm the transactions performed, and it has been designed to be a hard task. Using the concept of proof-of-work in order to provide a significant level of security to the Bitcoin network, the effort of validating Bitcoin information is rewarded, mainly, with the new bitcoins that are constantly created. Finally, the Bitcoin system needs to disseminate different kinds of information, essentially the payment transactions performed by users and the blockchain (or its actualization). The system transmits such information over the Internet through a distributed P2P overlay. Such a network is created by Bitcoin users in a dynamic way, and nodes of the Bitcoin P2P network are computers running the software of the Bitcoin network node. In regards to anonymity, Bitcoin achieves such a property by allowing users to create any number of anonymous Bitcoin addresses that will be used in their Bitcoin transactions.

Bitcoins are not digital objects, but an accounting entry in a Bitcoin account. Each Bitcoin account is identified by its Bitcoin address, and such an address is tied to an elliptic curve public key pair. Payments in the Bitcoin system are performed through transactions, which indicate the source address (the payer) and the destination address (the payee) of the Bitcoin payment. The payment destination can be determined only with the Bitcoin address, a public value known by any Bitcoin user, while the source of the payment is validated through a digital signature performed using the private key, a secret value only known by the owner of the source Bitcoin address.

Allowing multiple Bitcoin address generation in an anonymous way is a good starting point to achieve a good anonymity degree. However, the underlying non-anonymous Internet infrastructure used to propagate Bitcoin information, together with the availability of all Bitcoin transactions, has proven to be a potential anonymity threat, as different authors have pointed out [33,34]. However, as we will show later on, using existing tools like anonymous channels and Bitcoin mixing networks allows one to obtain a practical anonymous payment mechanism that can be applied to our MCS scenario.

### 3.1. Bitcoins as a Rewarding Mechanism

As a payment system, Bitcoin is a straightforward method to reward users in an MCS scenario. For each sensing value crowd sensors provide, they receive a Bitcoin payment as an awarded reward. Users can generate their Bitcoin addresses that will be used as rewarding addresses where the payer, probably the data collection center, using its own Bitcoin address, will send the Bitcoin payments for the sensing tasks the users perform.

Here, different properties of the Bitcoin currency system should be emphasized. First of all, bitcoins are suitable as a micro-payment system, since its eight decimal division provides a small enough granularity to define an efficient pay-per-sense model. On the other hand, Bitcoin payments do not need any intermediary between the payer and the payee, so the protocols for rewarding are straightforward since they do not involve any third party. Finally, it is worth mentioning that although fees applied to Bitcoin payments are lower than those in regular electronic payment systems (like credit cards), they are not low enough to use regular bitcoin payments as micropayments. Furthermore, despite the economic inconvenience of the fees, micropayments performed directly on standard Bitcoin payments are not recommended for efficiency reasons. Fortunately, Bitcoin allows one to create smart contracts where a micropayment channel can be established. Using such a channel, micropayments can be performed efficiently and with negligible fees.

On the other hand, when users’ privacy has to be taken into account, all mechanism used in the system have to provide a certain degree of anonymity. In this case, taking Bitcoin as the payment system to reward users is a good strategy due to the anonymity level provided by such virtual currency. As we already mentioned, Bitcoin anonymity is based on the easiness of anonymous Bitcoin addresses generation that will be used for the Bitcoin payments. For that reason, crowd sensors providing sensing data may generate multiple Bitcoin addresses, and the rewarding amount a user has to receive for all of his or her submitted reports could be spread over different Bitcoin addresses used for the rewarding payment.

### 3.2. Bitcoins as a Reputation Annotation Mechanism

As we already mentioned in Section 2.2, reputation measures may be used to assess the quality of the information that users send to the data collection center. Sensing data may be accepted or discarded based on the reputation value of the user reporting such data. Our approach is to adopt bitcoins also as a reputation annotation system, tying the concepts of reward and reputation. Such an approach could seem a limitation, since the unification of both concepts in a unique value implies that the reward system determines the reputation score and conversely. Nevertheless, from a conceptual point of view, reward and reputation concepts are closely related. Notice that reward for a given sensed value can be seen as a measure of the correctness of such a value, since reward should depend on the usefulness of that value. Following such an approach, the balance in a specific Bitcoin address will represent both the total awarded bitcoins for the sensing tasks reported with such Bitcoin address and the reputation obtained for the tasks.

Representing both concepts, reward and reputation, as the balance of a Bitcoin address has an interesting implication. For a Bitcoin address used with this purpose (reward and reputation), each further payment represents a withdrawal of economic funds, since bitcoins are transferred to another Bitcoin address, but also implies a reputation reduction. Conversely, users may desire to reduce their reputation in exchange for receiving some benefit. In that case, the associated withdrawal would be such a benefit. At first glance, it may not seem obvious the need for reputation reduction in an MCS scenario, but as we point out in Section 4.4, anonymous reputation schemes using multiple pseudonyms need to reduce users’ reputation in order to provide the unlinkability of pseudonyms.

## 4. The PaySense System

In this section, we describe PaySense, a Bitcoin-based system that provides an integrated mechanism for reward and reputation in MCS applications while preserving crowd sensors’ anonymity. The system can be seen as a plug-in for any MCS application that wants to reward its users to increase their engagement in the crowd sensing tasks. Furthermore, the MCS application also obtains from PaySense a mechanism to trace user’s reputation. As different proposals have been described [19], such a reputation value can be used to decide on the quality of the data that a user is reporting, allowing the MCS application to discard fake data provided by dishonest crowd sensors. Finally, the reward and reputation mechanism provided by PaySense comes also with a privacy guarantee. PaySense is a privacy-preserving system, so it is designed to keep users’ anonymity regarding the information involved in the PaySense system. However, a cautionary note on users’ anonymity has to be indicated when integrating PaySense with an MCS application. Depending on the specific data that the MCS application collects, the users’ identity may be revealed by different techniques. For instance, a user’s identity can be easily revealed through his or her position provided by the GPS sensor data, and other studies reported that only four spatiotemporal points are sufficient to identify users [22]. Such threads are beyond the PaySense anonymity measures and have to be mitigated using properly anonymizing techniques on the collected data, like those proposed by Shin *et al.* in [23].

### 4.1. PaySense Architecture Overview

**Figure 1:**
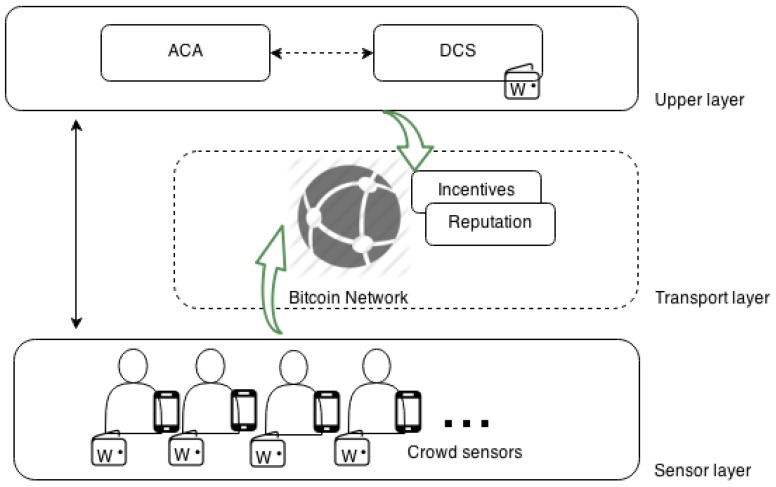
PaySense architecture and entities.

The architecture of a common MCS application comprises two main layers, as described by Ganti *et al.* in [35]: an upper layer where the sensed data are gathered and all of the processing takes places and a lower layer or sensor layer where the crowd sensor and all device-specific components reside. Then, all communications take place directly between these two layers. PaySense uses this same base architecture to facilitate integration and introduces a middle layer, the Bitcoin network, which provides a common framework to implement privacy-aware incentive and reputation models (see Figure 1).

In the PaySense architecture, each crowd sensor collects information about his or her surrounding environment following a participatory sensing paradigm and, then, sends the sensed data over conventional communication networks to the Data Collection Server (DCS). However, before sending sensed data to the DCS, crowd sensors must first obtain a Bitcoin address certificate from the Address Certification Authority (ACA), which enables them to receive rewards and reputation updates in the form of bitcoin payments for the sensing tasks they perform. Therefore, the Bitcoin network is integrated in the PaySense architecture as an additional transport layer for reputation and rewarding, while standard network communication channels are used for sensor data transfer.

### 4.2. PaySense Entities

The PaySense system is composed of different entities, some of them standard entities of any MCS system, and some others are specifically designed for the proposed system.

The **Crowd Sensors (CS)** are those entities in charge of sensing activities. Each crowd sensor, $CS_{i}$, collects data from his or her surrounding environment and sends them to the Data Collection Server (DCS). They are identified in the PaySense system through multiple Bitcoin certified addresses, that is $Addr_{i}^{j}$ for j=1,⋯,n. Note that the concept of a certified address does not exist in the Bitcoin system, but is a characteristicof PaySense as described in Section 4.3. Each Bitcoin address has an associated Elliptic Curve Digital Signature Algorithm (ECDSA) key pair, $\left(Pk_{i}^{j},Sk_{i}^{j}\right)$, and is constructed from the public portion of such a key pair as follows: $Addr_{i}^{j}=f\left(Pk_{i}^{j}\right)$ for a publicly-known cryptographic hash function f(·). Since Bitcoin addresses basically result in random alphanumeric characters, crowd sensors can use them as pseudonyms when communicating with other entities in order to preserve their anonymity. For that reason, we use both notations, namely Bitcoin address and crowd sensor pseudonym, indistinctly. Furthermore, the use of Bitcoin addresses allows crowd sensors to transact with other PaySense entities or even other Bitcoin users.

The **Address Certification Authority (ACA)** is the entity that certifies Bitcoin addresses so that those addresses could be accepted in the PaySense system. Each Bitcoin address $Addr_{i}^{j}$ for j=1,⋯,n owned by a crowd sensor $CS_{i}$ must be certified by the ACA in order to provide some degree of control over all existing users of the Bitcoin network and to avoid a Sybil attack. The Bitcoin address certificates, $Cert(Addr_{i}^{j})$, are issued following the *X.509v3* standard and with the ACA acting as issuing certification authority. The goal of the certification process is two-fold: on the one hand, it ensures that $CS_{i}$ cannot use more than one Bitcoin address at a given time, and on the other hand, it ensures that Bitcoin addresses are renewed periodically given the expiration date of the certificate, which actually limits the validity of the Bitcoin address itself . Although Bitcoin addresses do not expire, we apply the concept of expiration to the certificate issued by the ACA. When a certificate address expires, such an address cannot be used in the MCS system, but it is still a valid and usable standard Bitcoin address.

The **Data Collection Server (DCS)** is the entity that represents the MCS application server in charge of receiving and processing the sensed data sent by the crowd sensors. The DCS must perform a validation process on the received data and, based on its correctness, gives rewards to the crowd sensors providing such data. Rewards are provided through a Bitcoin transaction, and for that purpose, the DCS also holds a publicly-known Bitcoin address, $Addr_{DCS}$, and an ECDSA key pair associated with it. Such an address is publicly known by all crowd sensors, and it is used exclusively to reward crowd sensors for their readings through the Bitcoin network.

The **Bitcoin P2P network**, although not a real entity of the PaySense architecture, is used in the system to transfer bitcoins between PaySense certified Bitcoin addresses. Furthermore, the transaction information stored in the blockchain will be further used to assess the correctness of the PaySense system.

### 4.3. PaySense Interaction Model

In this section, we describe the different interactions between PaySense entities that are performed to execute all processes involved in our system.

#### 4.3.1. Crowd Sensor Enrollment

When a crowd sensor $CS_{i}$ wants to join the PaySense system for the first time, he or she must request a Bitcoin address certificate from the ACA by sending a certificate request, $CerReq(Addr_{i}^{j})$. As we already mentioned, the certificate is a standard X.509 certificate, so the certificate requests contain the address $Addr_{i}^{j}$ in the subject common name field. The real identity of the crowd sensor is required at this step in order to validate that he or she did not ask for another Bitcoin address certificate in the past trying to make a “fresh start” and preventing a Sybil attack. Then, the ACA verifies that the Bitcoin address has a *zero balance* (*i.e.*, does not contain any bitcoins) to ensure that $CS_{i}$ does not enter the system with a previously-assigned reputation score and issues a new Bitcoin address certificate $Cert(Addr_{i}^{j})$. Note that such a certificate guarantees that Bitcoin payments are being performed only for Bitcoin addresses owned by registered crowd sensors.

However, it is straightforward to notice that if $CS_{i}$ sends his or her $CerReq(Addr_{i}^{j})$ directly, then the ACA could link $Addr_{i}^{j}$ to $CS_{i}$’s real identity. To avoid this from happening, we adopt a blind signature scheme as follows:

1. $CS_{i}$ generates *n* different Bitcoin addresses (j=1,⋯,n) and for each one computes the certificate request, $CerReq(Addr_{i}^{j})$, and obtains its hash value, $h(CerReq\left(Addr_{i}^{j}\right))$.
2. $CS_{i}$ blinds each of the *n* hash values obtained in Step 1, $b_{i}^{j}=Blind(h(CerReq($ $Addr_{i}^{j})))$ for j=1,⋯,n, and sends the *n* hashed values to the ACA together with $CS_{i}$’s real identity. The blinding factor depends on the selected digital signature. Although it has been represented as a function for clarity, the blinding factor is a specific unrelated value for each different CerReq to blind.
3. The ACA randomly selects one of the received blinded hash values, namely $b_{i}^{k}$, and requests from $CS_{i}$ both the unblind factor and the $CerReq(Addr_{i}^{j})$ for the rest of the values j=1,⋯,k-1,k+1,⋯,n. Then, the ACA extracts the n-1 Bitcoin addresses contained in the received CerReq and verifies that all of them have a zero balance. Then, the ACA uses the unblinding factors to unblind each $b_{i}^{j}$ for j=1,⋯,k-1,k+1,⋯,n and checks that the unblinded values match with the hash values of the received CerReq.
4. If all verifications performed by the ACA in Step 3 hold, the ACA signs the blinded value $b_{i}^{k}$ and sends the result $Sig_{Sk_{ACA}}\left(b_{i}^{k}\right)$ to $CS_{i}$.
5. Upon reception, $CS_{i}$ unblinds the digital signature $Sig_{Sk_{ACA}}\left(b_{i}^{k}\right)$ performed by the ACA and uses the unblinded result together with the original $CerReq(Addr_{i}^{k})$ value to create the certificate: $Cert(Addr_{i}^{j})$.

Notice that although the ACA is signing a blind certificate, the cut-and-choose technique included in Step 3 ensures that a dishonest crowd sensor cannot obtain an arbitrary signature from the ACA with a better probability than $\frac{1}{n}$.

#### 4.3.2. Micropayment Channel Setup

In order to perform CSs rewarding, the DCS issues smart contracts [36] to every crowd sensor. A micropayment channel [37] is established with each one of those crowd sensors, allowing the DCS to pay for the users’ readings while avoiding paying high transaction fees.

The DCS sets up such micropayment channels by sharing a Bitcoin address with each user, where signatures from both parts are necessary to perform transactions. The micropayment channel is created during the CS enrollment and has the same expiration date as the CS certificate. New contracts will be issued after the address renewal.

#### 4.3.3. Sensing and Reporting Data

Once in possession of a certified address, crowd sensors can begin to report sensed data to the DCS. Prior to its transmission, the data are first digitally signed using the crowd sensor’s secret key, that is $sig_{data}=Sig_{Sk_{i}^{j}}\left(data\right)$, and later on, encrypted with the public key of the DCS. Then, each crowd sensor constructs the following sensing report: $Report=E_{PK_{DCS}}\left\{data,sig_{data},Cert\left(Addr_{i}^{j}\right)\right\}$.

Since the DCS is a central entity in the system, it is straight forward to provide his or her public key for encryption to all CSs of the system, using, for instance, the ACA during the crowd sensor enrollment. Notice, however, that the public key of a Bitcoin address is not a good choice as an encryption key since signing keys are strongly not intended for use together with encryption processes.

Although data encryption and the signature could imply some energy consumption in the crowd sensor device, in contrast to [38] where an SSL channel is established between the data sensor and the collection center, our proposal only encrypts and signs the sensed data, avoiding the overload that crypto-network protocols imply, for instance in the hand shake or in the shared key establishment. Furthermore, notice that our proposal uses elliptic curve cryptography, which deals with shorter keys and that has been demonstrated suitable for mobile devices [39].

Reports are sent over conventional communication networks to the DCS. Note that, our data-centric approach also allows the sensed information to pass through a multi-hop network, since no secure channel is required, but the source of such information could still be identified by the digital signature.

#### 4.3.4. Verification and Validation of Sensed Data

For each report received from a CS, the following validations are performed by the DCS:

1. Decrypts the report using his or her private key: $D_{SK_{DCS}}\left\{E_{PK_{DCS}}\left\{data,sig_{data},Cert\left(Addr_{i}^{j}\right)\right\}\right\}=\left\{data,sig_{data},Cert\left(Addr_{i}^{j}\right)\right\}$
2. Verifies that the data were sent by a registered crowd sensor. For that purpose, the DCS verifies the correctness of $Cert(Addr_{i}^{j})$.
3. Validates the source authenticity of the data by verifying the digital signature included in the report, that is $Ver_{Pk_{i}^{j}}\left(sig_{data}\right)=data$.
4. Applies a validation process on the data themselves to assess their quality.

If all validations are correct, the DCS provides a job reward and proceeds to update the reputation of the crowd sensor.

Notice that the data validation process performed in the last step is obviously application dependent. However, we assume that the reputation value of the crowd sensors who send the data will be involved in the validation process (*i.e.*, crowd sensors with a higher reputation are supposed to provide more accurate data). Such information is publicly stored in the Bitcoin blockchain, and the DCS can perform a crowd sensor reputation query (see the next interaction) to determine the level of trustworthiness of the crowd sensor and, consequently, the correctness of the contributed data.

#### 4.3.5. Crowd Sensor Reputation Query

The reputation value of a crowd sensor is equivalent to the sum of all of the DCS payments to that particular Bitcoin address. Furthermore, this information is publicly available in the blockchain database of the Bitcoin network. However, the DCS stores the current reputation of each CS. The purpose of such a store is two-fold: on the one hand, for efficiency, avoiding querying the blockchain for every CS reputation query and, on the other hand, for consistency reasons, because the actual reputation of a given CS is not updated in the blockchain until his or her current smart contract has ended.

In this way, the reputation of a PaySense user could be calculated as follows:

Reputation=transferred_reputation+earned_reputation-reputation_reduction

where earned_reputation refers to the reputation earned by the CS and paid by the DCS, transferred_reputation refers to the reputation transferred between the user’s pseudonyms and reputation_reduction refers to possible user punishment for misbehaving. The last two concepts will be shown in the *transfer reputation protocol*.

#### 4.3.6. Job Rewarding and Reputation Update

Once the sensed data have been validated, the user has to be rewarded and his or her reputation updated. Since in PaySense, both values are tied to a Bitcoin payment, the reputation update will determine the rewarding value. Again, the specific MCS application will define its suitable reputation model, which will determine the increasing amount of reputation score when users act properly in the system and provide correct readings. Once the increase on the reputation value has been established, the DCS transfers such reputation to the user by performing a micropayment using the micropayment channel previously established. The value of the payment will be the exact amount of reputation increment.

#### 4.3.7. Withdrawal of Rewarded Coins

At any time, crowd sensors can withdraw the bitcoins received as payments from the DCS, since there is no difference between a certified Bitcoin address and a standard one, from a transactional perspective. Nonetheless, it is important to stress that, according to the PaySense model of interaction, a withdrawal of funds implies a reduction in the reputation value associated with that Bitcoin address. Furthermore, it is worth mentioning that, as we will see in the *transfer reputation protocol*, the enforcing of withdrawal must be performed in order to provide unlinkability between pseudonyms. This reputation will become out of the system, but will be earned by the user as bitcoins.

#### 4.3.8. Transferring Reputation to a New Address

Before the Bitcoin address certificate expires, the crowd sensor has to obtain a new certificate from the ACA. Such certification renewal is especially sensitive because the Bitcoin address that is certified acts as a crowd sensor pseudonym. Then, in the process of transferring reputation to a new address, in order to provide a privacy-preserving mechanism, different constraints have to be meet:

- The ACA should not learn about the CS identity.
- The ACA should not link the old and the new address of a particular CS.
- The CS should not be able to increase his or her reputation.

From a CS real identity disclosure point of view, PaySense deals with those constraints by using a Bitcoin mixing process, described in the next section.

### 4.4. Transfer Reputation Protocol

Since PaySense uses Bitcoin addresses to store the reputation value for each CS, the reputation transfer is performed through a payment between Bitcoin addresses. However, due to the openness of the Bitcoin blockchain ledger, standard Bitcoin payments disclose both the sender and the receiver of a payment, and according to the constraints enumerated above, they are not suitable for a privacy-preserving reputation transfer.

In order to enhance the anonymity properties of the Bitcoin transactions and to use them for reputation transfer, we propose the use of mixing services, a procedure that shuffles the information in order to hinder the relation between the input and the output values of a transaction. The goal of Bitcoin mixing is to allow Bitcoin users to send bitcoins from one address to a mixing service and to receive from the mixing service the bitcoins to another address that could not be linked with the original one. Different mixing techniques have been proposed in the recent literature [40,41,42], each of them presenting different properties. For implementing PaySense, we chose the CoinJoin approach [40], since, although its main drawback is the need for a central server taking part in the mixing protocol, in our scenario, we already have such a central entity, since the ACA has to take an active part in the reputation transfer (issuing new certificates), so he or she can play the role of such a central server needed in the CoinJoin protocol.

The main idea of the PaySense *transfer reputation protocol* is to build a transaction that we call the *reputation transfer transaction*, where multiple CSs will transfer their reputations jointly from the old Bitcoin addresses to their new ones, hindering the link between input and output addresses. The greater the number of CSs involved in the protocol, the higher the anonymity reached.

In order to create the *reputation transfer transaction*, we use a method based on the CoinJoin mixing technique. CoinJoin takes advantage of the low level transaction structure using a multiparty protocol where different actors do not obtain any knowledge that could reveal the link between the input and output addresses of such a transaction. In order to understand the proposed protocol for the reputation transfer, we have to review some details of the Bitcoin transaction structure.

#### 4.4.1. Bitcoin Transaction Structure

**Figure 2:**
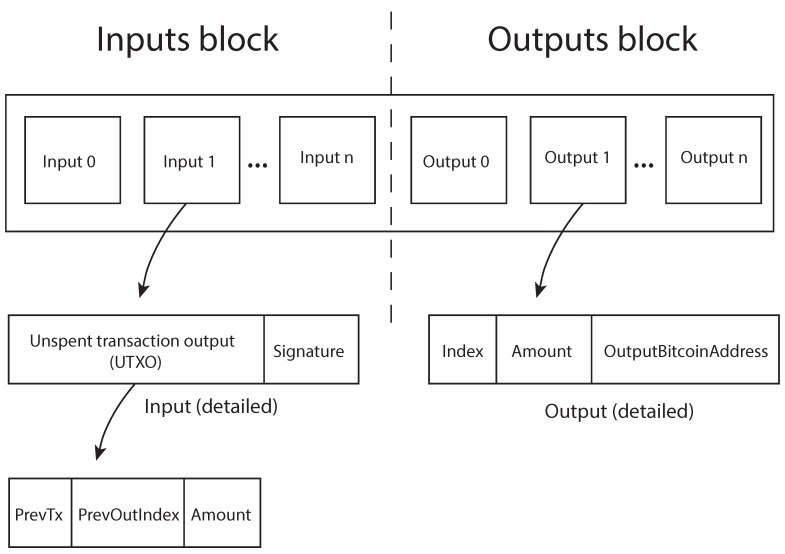
High level representation of a Bitcoin transaction.

Bitcoin payments are performed through Bitcoin transactions. A Bitcoin transaction is formed by two basic parts, the input block and the output block, as shown in Figure 2. In the simplest Bitcoin transaction, the input block and the output block contain exactly one single address.

When a user wants to perform a Bitcoin payment, he or she should build a transaction, including a set of non-spent previous transaction, also known as unspent transaction outputs (utxos), in the input block. The total Bitcoin amount of the inputs should be at least the amount to be spent. In addition, a list of outputs should be placed in the output block, representing the Bitcoin addresses to which the user wants to pay and the amount of bitcoins to be paid. The transaction input block represents the budget that the user has to spend and references the previous transactions where the money comes from, and the output block represents how the money is spent. Once the input and output addresses of the transaction are selected, the total transaction has to be signed. The standard Bitcoin transaction signature is performed by signing the transaction (inputs and outputs) with each private key related to the Bitcoin addresses where the funds come from; in other words, from each private key of the Bitcoin addresses referenced in the input block. For each input address, the signature is computed over the whole transaction value (inputs and outputs), and each signature is stored in the corresponding input of the input block as depicted in Figure 2.

Notice that with this procedure, no link could be established between transaction inputs and outputs, since each signature is performed over the same base information. Furthermore, the base information to be signed (inputs and outputs) has to be built before any transaction signature is performed.

#### 4.4.2. Transfer Reputation Protocol Description

Once the basic Bitcoin transaction format has been described, we can provide a high level description of our *transfer reputation protocol*. In such a protocol, different CSs create a *reputation transfer transaction* with the help of the ACA. First of all, every CS indicates his or her Bitcoin PaySense certified address as the input address and a new Bitcoin address as the output. Such a new address will be the new certified address for the new period. All of this information will be sent to the ACA, who would build the base information transaction, the data that each CS have to sign in order to validate the transaction. Then, the ACA sends the base information transaction to the CSs, and each of them performs the corresponding signature and returns the value to the ACA. Upon signature reception, the ACA builds the *reputation transfer transaction* with the base information transaction that he or she has created plus every signature received from the CSs and sends this to the Bitcoin network to be included into the blockchain. Finally, the ACA certifies all output addresses included in the *reputation transfer transaction*.

**Figure 3:**
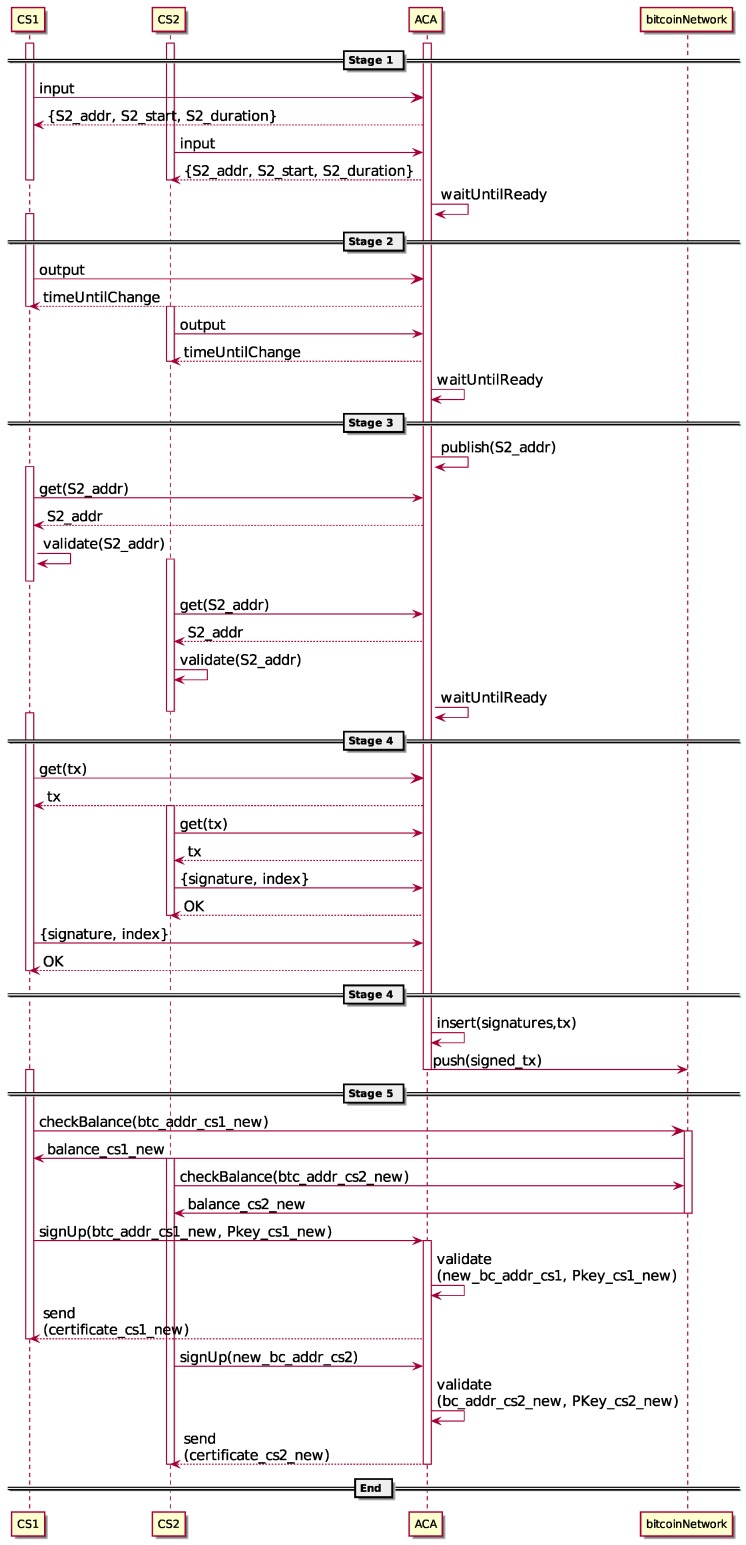
Transfer reputation protocol with two crowd sensors.

In Figure 3, the *transfer reputation protocol* is depicted. To avoid the disclosure of the link between the inputs and the outputs of the *reputation transfer transaction*, the ACA performs the data collection in five different and independent stages, recollecting different data in some of them. Moreover, the ACA is accessible to collect data from the CS only through the Tor network [43], being the web application offered by the ACA built as a Tor hidden service.

**Stage 1:** *Input recollection*. The first stage consists of recollecting the inputs of the *reputation transfer transaction*.

1. The ACA advertises a hidden service for transferring a predetermined and fixed reputation value *R* between certified Bitcoin addresses and new ones.
2. The ACA randomly generates, for later use, an output stage hidden service address, duration and starting time.
3. Crowd sensors with a certified Bitcoin address with reputation equal to or greater than the fixed *R* may join the protocol by sending a utxo with the exact value *R*. Notice that only one input per crowd sensor is allowed.
4. In case any CS does not have a single utxo of value *R* to send to the mixing service, he or she could perform an *reputation unifying transaction* to create it (described in the next subsection). The new generated utxo will be sent as the input.
5. The ACA verifies that each crowd sensor trying to participate in the protocol has at least a reputation score of *R*, including possible reputation reduction punishment for misbehaving.
6. The ACA verifies that each received input comes from a valid source. There are only three valid sources: the DCS, a previously-certified Bitcoin address and the same address itself. Transactions from the DCS are bound to the *earned reputation* that have been paid by the system to the user. Transactions from a previous certified Bitcoin address are bound to *transferred reputation* and are limited to just one per address (the very first one actually). Finally, transactions from the same address are bound to a *reputation unifying transaction* (see the next subsection for details).
7. The ACA sends the output stage hidden service address, duration and starting time to each CS whose inputs have been accepted.
8. The ACA ends such an input recollection stage after a predefined time discarding any input with a value different from *R*.

**Stage 2:** *Output recollection*. In the second stage, all outputs of the *reputation transfer transaction* are collected.

1. Crowd sensors that have already sent the input in the previous stage now send the output. Again, only a single output for each crowd sensor is allowed.
2. The ACA validates that all of the outputs have an exact value of *R*.
3. The ACA, using the inputs and outputs received in the first and second stages, constructs the base non-signed *reputation transfer transaction*.
4. The ACA ends the output recollection stage after a predefined time.

**Stage 3:** *Address commitment*. In the third stage, the ACA openly publishes the output stage hidden service address.

- The ACA publishes the output stage hidden service address he or she has sent to the participating CS in Stage 1.
- Each CS could now check that the previously-provided output stage hidden service address matches with that openly published.
- The ACA ends the stage after a predefined time.

**Stage 4:** *Signature recollection*. In the fourth stage, the *reputation transfer transaction* is distributively signed.

1. Crowd sensors request the base non-signed *reputation transfer transaction* from the ACA.
2. Crowd sensors sign the obtained value and send the result to the ACA.
3. The ACA checks the correctness of every provided signature.
4. The ACA composes the *reputation transfer transaction* with the base non-signed transaction that he or she built in the second stage and each of the signatures received from each crow sensor.
5. Finally, the ACA pushes the resulting *reputation transfer transaction* to the Bitcoin P2P network to be included in the blockchain.

**Stage 5:** *Address certification*. In the last stage, the ACA generates and publishes the new certificates corresponding to the output addresses of the *reputation transfer transaction*.

1. Crowd sensors validate that the *reputation transfer transaction* has been published in the blockchain and that his or her reputation has effectively been transferred to the new Bitcoin address.
2. Crowd sensors send to the ACA the public key corresponding to the Bitcoin address where the reputation has been transferred.
3. The ACA verifies that the received public key matches one of the Bitcoin addresses included as output in the *reputation transfer transaction* and that the address has only one payment related to it. If the validation is correct, he or she sends the new certificate to the crowd sensor.

#### 4.4.3. Reputation Unifying Transaction

As described in the Bitcoin protocol, *utxos* must be spent entirely each time a transaction is performed. This makes it especially complicated to find a unique *utxo* that matches the amount of reputation that a certain user wants to transfer to their new pseudonym. Moreover, taking into account that reputation will be passed though multiple pseudonyms over time, finding a suitable *utxo* is even harder. In order to solve this, CSs will perform a previous step to unify the desired amount of reputation. To doing so, a CS willing to perform a reputation exchange will create a transaction being himself or herself (his or her certified Bitcoin address) both the source and destination of the transaction, using the necessary *utxos* to reach the desired amount of reputation. The newly-generated *utxo* will be the chosen one in the *transfer reputation protocol*. It is important to stress that in order to provide unlinkability and since multiple crowd sensors will act together to build the *reputation transfer transaction*, every crowd sensor has to reduce his or her reputation a bit, to an amount that matches *R*, as pointed out in the *reputation transaction protocol*.

### 4.5. PaySense Integration with Existing MCS Systems

As we already pointed out, PaySense is proposed as a plug-in for any MCS application that wants to reward its users to increase their engagement in the crowd sensing tasks. For that reason, its implementation is tied to a main MCS application to which PaySense will be integrated. PaySense extends the functionality of the two main actors of the MCS application, which are: the DCS and the CSs; and also including a new actor responsible for address certification and involved in the *transfer reputation protocol*: the ACA.

The first modification needed in an MCS system to allow a PaySense integration is that CSs have to be identified only by means of a bitcoin address. This main property may affect how the DCS stores CSs information and interactions and also how CSs deal with its identity.

Secondly, for the PaySense integration, the DCS needs to run a Bitcoin wallet in order to reward users’ reports with bitcoins. A wide range of wallets could be deployed within the DCS; however, we suggest the reference implementation client [44], since it is endorsed by the Bitcoin Core developers. Payments performed with this wallet should replace the rewarding mechanism of the MCS system. Furthermore, integration of PaySense in the DCS system also involves the data validation process. In order to obtain reliable data, the DCS should apply a validation process to all of the users’ reports. PaySense suggests to use the reporter’s reputation in such a process, a value that is provided to the MCS system through the *crowd sensor reputation query interaction* (see Section 4.3.5 for details) and that can be used to create or complement the internal validation process that the MCS has defined.

As far as CSs are concerned, two main functionalities are needed in order to integrate PaySense: a Bitcoin wallet and a Tor network connection. The wallet is needed to create the CS pseudonym, the bitcoin address and to check the benefit obtained from the reported sensing. The wallet also allows one to transfer reputation between addresses and to cash some (or all) received bitcoins. Different types of wallets could be used depending on the resource restrictions of the CS, from the reference implementation client, to Simplified Payment Verification (SPV) clients, such as bitcoinj [45] or Electrum [46]. The Tor connection will be necessary to interact with the ACA during the *transfer reputation protocol*, since the service is only available through the anonymization network. Any CS’s pseudonym modification restriction may be disabled from the original application, as PaySense identifies his or her CSs with Bitcoin addresses previously certified by the ACA, and the change of the pseudonym is a system requirement in order to preserve users’ anonymity. In addition, every CS may include a module to perform ECDSA signatures and to encrypt messages to report data to the DCS.

Finally, the ACA should be deployed as a new server entity separate from the DCS. The ACA has the core capabilities of a traditional Public Key Infrastructure (PKI) certification authority, such as certificate generation, maintenance and revocation, but it also takes an important role in the *transfer reputation protocol*. In such a protocol, the ACA builds Bitcoin transactions, but instead of standard generation, it acts as a mixer without being involved with its own addresses. All of those protocols in which the ACA takes part have been provided as an open source Python implementation [6] and comes along with a Python utils package [47].

## 5. Security Analysis

In previous sections, we have described the PaySense functionality showing how different entities that are part of the system interact with each other, providing different kinds of information and performing a wide range of tasks that conform to the PaySense functionality. However, some of the actors could misbehave during the system lifetime; the reason why every interaction should be as secure as possible. In this section, we describe our adversary model, and we analyze how the system provides a high security level in relation to different attacks.

### 5.1. Adversary Model

Our adversary model can be divided into two different categories: external and internal attackers. On the one hand, an external attacker is an attacker that is not part of the system, but tries to disrupt its correct functionality. As an external entity, we assume that he or she has no internal information; mainly, he or she does not have a certified Bitcoin address by the ACA. However, the external attacker may have standard Bitcoin addresses that may contain some balance. On the other hand, an internal attacker could be either a CS, the ACA or the DCS. As we will analyze later, the objectives of an internal attacker differ depending on his or her role in the system. Furthermore, notice that an internal attacker is, obviously, more powerful than an external one. In fact, all possible attacks described by an external attacker can be performed by an internal one, from the point of view of the knowledge needed to perform the attack.

Next, we enumerate the possible objectives of an external attacker:

1. **CS identity disclosure** aims to discover the identity of a certain user. It could be divided into two degrees depending on the level of disclosure: full disclosure, where the pseudonym is linked with the user’s identity, and pseudonym linkability, where two or more pseudonyms are known to belong to the same user.
2. **Certificate tampering** consists of the inclusion of fake data inside a certificate to get some additional gain during the registration.
3. **The CS data sensing tampering** objective is to generate fake data and to try to pass it off as correct or to modify some data originally created by some user in such a way that it becomes false.
4. **The Sybil attack** provides a single attacker with more than one pseudonym in the system, that is multiple identities.
5. **The DoS attack** consists of interrupting the normal system behavior. Traditionally, general DoS attacks are usually related to low level implementation details or integration specificities. We do not intend to address this kind of attack, but those DoS attacks related to high level protocol dysfunctions.

The objectives of internal attackers depend on the attacker role. Objective attacks for the CS are the following:

1. **A fraudulent reputation increase** is a group of attacks where an attacker tampers with the information provided to the system in order to obtain more reputation than the supposed one. Four attacks fit inside this group: fake reward attack, target reputation attack, stale address attack and reputation injection attack.
2. **Steal rewards** consist of changing the reported data in such a way that it looks like the attacker is the original issuer, and therefore, the reward is assigned to him or her.
3. **Data sensing poisoning** aims to send fake information to the DCS in order to spread this information over the network taking some advantage with it.
4. **A fraudulent reward increase** occurs when a certain reward is paid more than once to the same user for the same provided data.

CS also shares the following objectives with external attackers:

5. CS data sensing tampering.
6. Sybil attack.
7. DoS attack.

Finally, we assume that both the DCS and the ACA act honestly, since they are the main running parties of the system, and most of the attacks envisaged so far do not make sense. We assume that the dishonest behavior from those entities is restricted to an identity disclosure attack, both trying to learn either the real identity of the CS or the linkability of his or her pseudonyms.

In order to simplify the analysis, when the same objective is pursued by different entities, we perform a single analysis using the most restrictive case. For instance, an attack trying to disclose the CS identity can be performed by the ACA, by the DCS, by another CS and by an external attacker. Since the ACA is the entity that has more information from the process of enrollment and reputation transfer, ensuring that the ACA cannot disclose the CS identity extends such a property to other entities that have less information to perform the same attack.

### 5.2. Security Measures

The first attack we analyze is the **CS identity disclosure**. As we already mentioned, this attack can be divided into two different objectives. Disclose the real identity of the CS or provide linkability across multiple pseudonyms (certified addresses) of the CS. The entity with the most privileged position in trying to disclose users’ identity is the ACA.

The ACA could try to link a pseudonym with the real user’s identity during the process of enrollment, since it is the only time both attributes (real identity and CS pseudonym) are provided together. Nonetheless, the CS will end up using the only pseudonym from the *n* provided ones, which the ACA does not know anything about, since the ACA will perform a blind signature to produce the certificate that contains it.

Pseudonym linkability, on the other hand, could be attempted by the ACA during the *transfer reputation protocol*, since the process requires CSs to provide their current and new pseudonyms. Nevertheless, all communications between the ACA and every CS are performed using a Tor anonymous network and hidden services; then, the ACA will not succeed in such an attack unless a CS makes unappropriated usage of the network.

At first glance, this should be enough to avoid a malicious ACA from learning about users’ identity. Unfortunately, it is not. If the ACA misbehaves, he or she could send different server addresses to each of the participants, linking in this way the inputs with the outputs of the *reputation transfer transaction* and, if the transaction is finally signed and published, being able to disclose the link between old and new CS pseudonyms. PaySense deals with this kind of threat by forcing the ACA to commit to a random, unique and verifiable output stage hidden service address for each *reputation transaction protocol* instance. If any of the participants notices that both addresses do not match, he or she could refuse to sign the transaction, forcing it to be invalid, and therefore, rejected by the Bitcoin network. It is important to stress that even a link between pseudonyms could be built by a misbehaving ACA after the output stage, he or she will end up learning nothing at all, since the transaction will not be published, and new pseudonyms will be freshly generated for a future reputation transaction.

Launching a **certificate tampering attack** is another way for a malicious user to achieve some non-earned reputation. In such an attack, the attacker may try to insert any kind of fake information inside the certificate to be signed, taking advantage of the fact that the ACA performs a blind signature. Even though modifying some X.509 certificate fields could be detrimental for the CS, such as including an invalid issuer, other information could give him or her some advantage over the rest of the users. Using a non-fresh Bitcoin address is a perfect example of this. If an attacker provides a Bitcoin address containing some balance, he or she could end up being enrolled in the system with non-zero reputation. While this is a valid attack that a malicious user can try to perform, the success provability can be made arbitrarily low. An attacker performing such an attack will only succeed with a probability of 1/n, being *n* the total number of certificates requested by the ACA during the registration process. Moreover, since the user’s identity is provided during the process, if a user is caught up trying to perform such an attack, he or she could be banned from the system.

**CS data sensing tampering** is a different attack of which to be aware. It is based on a well-known privacy disclosure attack, the Man in the Middle (MiM). The attack consists of a modification of the data sent by a certain CS to the DCS in a way that the information provided becomes false. The main objective of such an attack is to undermine the reputation of the targeted CS. As long as the information provided by the CS to the DCS is digitally signed by his or her issuer, the DCS would not accept the information, and the attack will not succeed.

An attacker may try to launch a **Sybil attack** to obtain an additional pseudonym. Even though the system is specially designed to avoid such attacks, an incorrect usage from the users could give an attacker an opportunity to do so. The only way for an attacker to perform such an attack is through an incorrect management of another user’s keys. As long as an attacker is able to steal another user keys, he or she could not only steal all of the funds in the account related to it, but also obtain an additional pseudonym by performing a *transfer reputation protocol*. If the attacker could get a new pseudonym from a reputation transfer before the real user could notice the key was stolen, that pseudonym will be untraceable according to the unlinkability property of such a process. This kind of problem comes from the Bitcoin transaction irreversibility, and it is inherent to the Bitcoin design. If a user loses his or her keys, there is nothing to do. Proper key management is required when Bitcoin comes into play.

We will follow the analysis by evaluating the possible **Denial of Service (DoS)** attacks. To do so, we should consider which points of the system are potentially points of failure, or, the same, the attack of which parts of the system will result in an interruption of normal PaySense behavior. Following this approach, the most conflictive point is the one where more actors interact together: the *transfer reputation protocol*.

From a security analysis perspective, the main drawback of the *transfer reputation protocol* is that the number of inputs, outputs and signatures, provided at different stages of the protocol, must match in order to build and broadcast the *reputation transfer transaction*. Otherwise, all of the data are discarded, and no reputation is transferred.

The input recollection stage being our first point of analysis, it is straightforward to notice that no tampering could be performed, since the ACA will discard any non-valid input after being checked. On the contrary, the output recollection is easier to attack, as it is expected that only users who have sent inputs to the previous stage send the corresponding output in the next stage. Any user could try to send a random output resulting in a non-matching number of inputs and outputs. If this situation is detected by the ACA, the transaction will be conclusively discarded. To avoid suck an attack, PaySense randomizes the output stage starting time, duration and the server address where it will take place, limiting the ability to perform the attack to those involved in the input stage. Using this approach, an address involved in the input stage of different protocols’ performances that has suffered a DoS attack could be banned.

A similar DoS attack could be used in the signature recollection stage, since again, no link is established between users participating in previous stages, allowing any user to send fake signatures to the ACA. However, no threat is presented through this approach, since the ACA could use the digital certificates associated with every Bitcoin address to check the correctness of each signature before including it into the transaction (notice that the ACA knows where each signature has to be placed and which CS has participated in the protocol). Therefore, duplicated and wrong signatures will be discarded. Finally, a malicious user could force the transaction to be discarded by not sending his or her signature. However, the malicious user will be automatically pointed out and, therefore, punished for it.

Following the approach of information tampering, a **fraudulent reputation increase** is a considerable big group of attacks aiming to provide wrong information to the system in order to obtain some amount of non-earned reputation. As mentioned before, four different attacks fit inside this group: fake reward attack, target reputation attack, stale address attack and reputation injection attack.

First of all, a CS could try to perform a fake reward attack by performing payments from addresses outside the system to his or her certified address, in order to increase his or her reputation. Since, in the *transfer reputation protocol*, the reputation is computed using only transactions from a certified address, or from the DCS, the reputation score of a CS cannot be increased using such an attack.

A CS could also try to increase his or her reputation through a target reputation attack, by participating in a *transfer reputation protocol* with a reputation target *R* higher than his or her actual reputation. Since the ACA checks all of the provided inputs of the *transfer reputation protocol*, the attacker’s input will be rejected.

Moreover, a CS could try to take advantage of a stale address attack to increase his or her reputation fraudulently. To do so, he or she could send a non-freshly-generated Bitcoin address to the output stage of the *transfer reputation protocol* to earn the balance associated with that address as reputation during the certification process. Since the ACA checks that every address sent to the output recollection stage has zero balance, the output will be discarded, and the attack will not succeed.

A variation of the previous attack, the reputation injection attack, could also be performed by a malicious CS. In this case, a freshly-generated Bitcoin address will be sent to the output recollection stage, but a transaction will be performed from another address to that one before the certification process. Even in this case, the address will not be certified since the ACA checks that the address to be certified has only one *utxo* related to it, and that *utxo* belongs to a *reputation transfer transaction*. Moreover, since the address will not be certified, but the reputation transfer transaction would be published, the reputation transferred to the attackers’ address will became out of the system permanently.

**Steal rewards** is a different kind of attack where a malicious user aims to obtain some non-earned reputation. It is similar to the previously-introduced CS data sensing tampering, but its purpose differs. The attack lies in a malicious user changing the issuer field of a report to himself or herself (his or her certified pseudonym) and performing a new signature over the data as if he or she were the original issuer. In this way, the possible rewards for the data sensing and reporting will be assigned to the attacker instead of the original issuer. PaySense deals with this kind of data modification attack by encrypting the communications between CSs and the DCS, since every user knows the DCS public key.

Following a different approach, **data sensing poisoning** could be used to achieve two of the previously pointed out objectives together: increasing the reputation of one or more CS and providing the wrong information to the system to take some external advantage. In order to perform this attack, a group of users should agree in reporting the same kind of fake information. If the number of reporters is big enough, the data could end up being accepted and the attackers rewarded for it. Protection against this type of attack depends on the data validation procedure applied (see Section 4.3.4), and as we already mentioned, it is application dependent. PaySense provides a reputation system for applications to validate information and to avoid such kinds of attacks. The interested reader could refer to [19] for detailed information of the data validation through users’ reputation.

Moreover, if an attack like this is detected by the DCS, malicious users will be punished with a reputation reduction. Hence, each time an attack is detected, the power of malicious users is reduced.

Finally, a **fraudulent reward increase** is the last attack of our analysis. As in the steal rewards attack, the attacker aims to obtain some non-earned reputation, but in this case, it is not stolen from another user, but obtained by duplicating readings. However, the DCS will not reward duplicated reports (*i.e.*, same information from the same issuer), so the attack will not succeed.

## 6. Conclusions and Further Research

With MCS arising as a new sensing paradigm, new singular research challenges are introduced, such as fostering participation among the crowd sensors, ensuring the trustworthiness of the contributed data since counterfeit information may be contributed by malicious individuals and, at the same time, preserving the anonymity of the crowd sensors.

We have presented PaySense, a framework that addresses in a practical way all of the aforementioned challenges together, using the Bitcoin network as an integrating solution. Crowd sensors contribute sensed data using certified Bitcoin addresses and reputation and rewards are mapped to a single bitcoin funds transfer, which can later be spent by their owners. Using such an approach, our system inherits the privacy-preserving properties that bitcoins present. As a main contribution, our proposal solves the problem of reputation transfer satisfactorily when dealing with anonymous scenarios. Previous proposals impose a reduction of each user reputation when transferring the reputation between pseudonyms without any other benefit than preserving unlinkability between pseudonyms. In our solution, pseudonym unlinkability comes for free since the reduction of the reputation is transformed in an economical profit thanks to the fact that reputation is expressed directly in bitcoins.

Further extensions of the proposed system may be directed to enable a single ACA shared by multiple MCS applications or even by other applications outside the field of MCS, enabling users to create a global digital reputation score useful in multiple environments. Furthermore, further research has to be directed toward analyzing the behavior of different reputation models when reward and reputation are considered as a whole.
